# Crystal Structure of the Monomeric Extracellular Domain of α9 Nicotinic Receptor Subunit in Complex With α-Conotoxin RgIA: Molecular Dynamics Insights Into RgIA Binding to α9α10 Nicotinic Receptors

**DOI:** 10.3389/fphar.2019.00474

**Published:** 2019-05-01

**Authors:** Marios Zouridakis, Athanasios Papakyriakou, Igor A. Ivanov, Igor E. Kasheverov, Victor Tsetlin, Socrates Tzartos, Petros Giastas

**Affiliations:** ^1^Department of Neurobiology, Hellenic Pasteur Institute, Athens, Greece; ^2^Institute of Biosciences and Applications, NCSR “Demokritos”, Athens, Greece; ^3^Shemyakin-Ovchinnikov Institute of Bioorganic Chemistry, Russian Academy of Sciences, Moscow, Russia; ^4^Institute of Molecular Medicine, Sechenov First Moscow State Medical University, Moscow, Russia; ^5^PhysBio of MEPhI, Moscow, Russia; ^6^Department of Pharmacy, University of Patras, Patras, Greece

**Keywords:** nicotinic acetylcholine receptors, α-conotoxins, RgIA, structure, molecular modeling, molecular dynamics

## Abstract

The α9 subunit of nicotinic acetylcholine receptors (nAChRs) exists mainly in heteropentameric assemblies with α10. Accumulating data indicate the presence of three different binding sites in α9α10 nAChRs: the α9(+)/α9(−), the α9(+)/α10(−), and the α10(+)/α9(−). The major role of the principal (+) side of the extracellular domain (ECD) of α9 subunit in binding of the antagonists methyllylcaconitine and α-bungarotoxin was shown previously by the crystal structures of the monomeric α9-ECD with these molecules. Here we present the 2.26-Å resolution crystal structure of α9-ECD in complex with α-conotoxin (α-Ctx) RgIA, a potential drug for chronic pain, the first structure reported for a complex between an nAChR domain and an α-Ctx. Superposition of this structure with those of other α-Ctxs bound to the homologous pentameric acetylcholine binding proteins revealed significant similarities in the orientation of bound conotoxins, despite the monomeric state of the α9-ECD. In addition, ligand-binding studies calculated a binding affinity of RgIA to the α9-ECD at the low micromolar range. Given the high identity between α9 and α10 ECDs, particularly at their (+) sides, the presented structure was used as template for molecular dynamics simulations of the ECDs of the human α9α10 nAChR in pentameric assemblies. Our results support a favorable binding of RgIA at α9(+)/α9(−) or α10(+)/α9(−) rather than the α9(+)/α10(−) interface, in accordance with previous mutational and functional data.

## Introduction

Nicotinic acetylcholine receptors are the prototypic members of the Cys-loop family of pentameric ligand-gated ion channels, including also the 5-HT_3_, GABA_A_, and glycine receptors (Lester et al., 2004; Sine and Engel, 2006; Albuquerque et al., 2009; Nemecz et al., 2016). Muscle and neuronal nAChRs are found at the neuromuscular junction and in central and peripheral neurons, respectively. Neuronal nAChRs regulate neuronal excitability and neurotransmitter release and, in humans, are composed of a combination of eight α (α2–7, α9–10) and three β (β2–4) subunits, forming either homopentamers or heteropentamers (e.g., α7, α4β2, α7β2, and α9α10 nAChRs) (Albuquerque et al., 2009; Millar and Gotti, 2009; Engel et al., 2015). These receptors are also found in the immune system and in various peripheral tissues (Wessler and Kirkpatrick, 2008; Beckmann and Lips, 2013). Each neuronal nAChR subtype has distinct pharmacological and electrophysiological properties and distinct localization within the central and peripheral nervous system (Gotti et al., 2006, 2007; Millar and Gotti, 2009; Taly et al., 2009).

Due to their implications in smoking addiction and in various neurological and non-neurological diseases and disorders (e.g., Alzheimer’s and Parkinson’s diseases, schizophrenia, neuropathic pain, and inflammation), neuronal nAChRs are important drug targets (Taly et al., 2009; Quik et al., 2011; Dineley et al., 2015; Hone and McIntosh, 2018). However, due to the high similarity in the orthosteric ligand-binding site of neuronal nAChRs, the development of drugs targeting specifically a distinct nAChR subtype is a very challenging task, requiring detailed structural information. This site consists of loops A, B, and C of the principal (+) side of the ECD of an α subunit and of loops D, E, and F of the complementary (−) side of the ECD of the adjacent α or β subunit (Brejc et al., 2001; Unwin, 2005). Whereas the highly conserved (+) side of the ligand-binding site seems to play an important role in the orientation of the bound ligand (Dellisanti et al., 2007; Zouridakis et al., 2014), it is the less conserved (−) side that determines the selectivity on a specific nAChR subtype (Rucktooa et al., 2009; Bourne et al., 2015; Giastas et al., 2018).

Apart from the early breakthrough cryo-electron microscopy studies of the *Torpedo* muscle-type nAChR (Unwin, 1995, 2005), our understanding of the structure of neuronal nAChR ligand-binding sites was greatly advanced by the X-ray crystal structures of the molluscan AChBPs (Brejc et al., 2001; Celie et al., 2004). AChBPs share up to 24% sequence identity with the ECDs of nAChRs and, most importantly, almost all residues that are conserved in the ligand-binding sites of nAChRs are also found in them. In addition, the X-ray structures of mutated or chimeric AChBPs, carrying single-point mutations or region replacements, respectively, to mimic some nAChR-ECDs, have shed light on the structure of the ligand-binding sites formed between α7 or α4 or between α3 and β4 nAChR subunits (Li et al., 2011; Nemecz and Taylor, 2011; Shahsavar et al., 2015; Abraham et al., 2017). Recent breakthrough X-ray and electron microscopy studies of the almost intact α4β2 nAChR (Morales-Perez et al., 2016; Walsh et al., 2018) with nicotine bound, elucidated the 3D structure of α4(+)/α4(−) and α4(+)/β2(−) binding sites in high detail. Also, the crystal structure of the α2(+)/α2(−) binding site, present in α2β2 nAChRs, was solved previously with the agonist epibatidine bound (Kouvatsos et al., 2016), succeeding the crystal structure of the monomeric α9-ECD in its free and antagonist [methyllylcaconitine (MLA) or α-bungarotoxin (α-Bgtx)]-bound forms (Zouridakis et al., 2014).

Subtype-specific inhibitors of nAChRs, apart from valuable tools for dissecting the roles of the various nAChRs, may also be important therapeutic agents. A good example of subtype-specific nAChR antagonists are α-conotoxins (α-Ctxs), peptides isolated from the venom of snails belonging to the *Conus* genus (see reviews Nicke et al., 2004; Janes, 2005; Azam and McIntosh, 2009; Rucktooa et al., 2009; Tsetlin et al., 2009). α-Ctxs range in size from 12 to 19 amino acid residues and contain two disulfide bonds with a Cys1-Cys3 and Cys2-Cys4 connectivity, forming two backbone loops (loop 1 and loop 2, respectively). RgIA, on which the present study is focused, is a 4/3 subclass α-Ctx (containing four and three residues in loops 1 and 2, respectively), and is highly selective for the α9α10 nAChR (Ellison et al., 2006, 2008; Clark et al., 2008). This neuronal nAChR subtype has two stoichiometries, the (α9)_2_(α10)_3_ and (α9)_3_(α10)_2_ (Plazas et al., 2005; Indurthi et al., 2014), and is mainly expressed in the hair cells of the cochlea (Elgoyhen et al., 1994, 2001) and in a variety of immune cells (Lustig et al., 2001; Peng et al., 2004; Hecker et al., 2015). Mutational and functional data suggest that there are three ligand-binding sites in α9α10 nAChRs, namely the α9(+)/α9(−), α9(+)/α10(−) and the α10(+)/α9(−) (Ellison et al., 2008; Indurthi et al., 2014; Azam et al., 2015; Boffi et al., 2017).

There are many X-ray structures of different AChBPs in complexes with various α-Ctxs, but here we present the first X-ray crystal structure of an α-Ctx (RgIA) bound to the nAChR α9-ECD, solved at 2.26-Å resolution. As the protein is in a monomeric state, the interactions of RgIA with the (+) side of the α9-ECD were revealed and were found to be similar to those between the (+) side of AChBP protomer and, the similar to RgIA, α4/3-Ctx ImI (Hansen et al., 2005; Ulens et al., 2006). Moreover, RgIA in its complex with α9-ECD superimposed very well with other α-Ctxs bound to pentameric AChBPs, denoting that the α9(+) side in α9α10 nAChRs is instrumental for the orientation of the bound RgIA, similarly to what was previously shown for MLA and α-Bgtx binding to α9-ECD (Zouridakis et al., 2014). In addition, based on the crystal structure of α9-ECD/RgIA, MD simulations of the complexes of RgIA at α9(+)/α9(−), α9(+)/α10(−), and α10(+)/α9(−) binding sites in human nAChR α9α10-ECDs were performed. These studies indicated that the favorable binding sites for RgIA are the α9(+)/α9(−) and/or α10(+)/α9(−), rather than the α9(+)/α10(−), in accordance with previous mutational and functional data. Since the α9α10 nAChR is a possible pharmacotherapeutic target for the auditory disease tinnitus and for the chronic neuropathic pain (Elgoyhen et al., 2009; Elgoyhen and Langguth, 2010; Hone and McIntosh, 2018), this study may provide useful information for the design of highly selective improved RgIA analogs for use against such diseases.

## Materials and Methods

### Materials

The materials and reagents used for purification and deglycosylation of nAChR α9-ECD were Ni^2+^-NTA resin (Qiagen, United States) and EndoH_f_ (NEB, United States). All other chemicals used were of analytical grade (SIGMA-ALDRICH, United States). For the solid-phase peptide synthesis of α-Ctx RgIA, we used: Fmoc-protected amino acids and diisopropylcarbodiimide (Iris Biotech GmbH, Germany), a block-copolymer resin Tentagel HL-NH2 modified with Knorr linker (Tentagel-RAM, Rapp Polymere, Germany), 4-methylpiperidine (Acros Organics, Belgium), hydroxybenzotriazole (Riyn Group, China), trifluoroacetic acid (Solvay Chemicals, Belgium). Other reagents were acquired from local supplier. All reagents and solvents were used without additional purification.

### Protein Expression and Purification

The human nAChR α9-ECD was expressed as a C-terminal six-histidine tagged protein in the yeast *Pichia pastoris* system and purified by metal affinity and size exclusion chromatography (SEC); enzymatic deglycosylation was carried out with endoglycosidase EndoH_f_, as also described in Zouridakis et al. (2014).

### Ligand-Binding and Competition Experiments

Ligand-binding experiments to test [^125^I]α-Bgtx binding to the glyco- and deglycosylated α9-ECD were described and performed previously (Zouridakis et al., 2014). The K_d_ values of [^125^I]α-Bgtx for the glyco- and deglycosylated protein were 30 and 19 nM, respectively (Zouridakis et al., 2014). Competition experiments of [^125^I]α-Bgtx binding to the α9-ECD by α-Ctx RgIA were performed with SEC-purified monomeric glycosylated or deglycosylated histidine-tagged α9-ECD bound to Ni^2+^-NTA beads. The beads were washed twice with 10 volumes of phosphate buffer saline (PBS) and then diluted 10 times with PB-BSA buffer [10 mM potassium phosphate buffer, 0.2% bovine serum albumin (BSA), 0.05% NaN_3_, pH 7.4]; 10 μl of this dilution were used in each reaction. The protein concentration was 100 nM and the specific activity of [^125^I]α-Bgtx was 30 cpm fmol^−1^. Reaction volume was fixed to 50 μl by addition of PB-BSA buffer. Competition experiments were performed with fixed [^125^I]α-Bgtx concentration at 50 nM and variable unlabeled RgIA concentrations (1 nM–200 μM). Serial dilutions of the stock buffer of RgIA (initial concentration of 4.8 mM) were done in PB-BSA buffer. Incubation of reaction mixtures was performed overnight at 4°C. The beads were then washed three times in 1 ml of 20 mM Tris and 0.05% Triton X-100, pH 7.5, followed by a final centrifugation at 1000 *g*, 5 min at 4°C. Finally, the bound radioactivity was measured on a gamma counter. Non-specific binding was measured in samples with the same ingredients but without the α9-ECD. All assays were performed in triplicate, and binding data were evaluated with an algorithm of GraphPad Prism 5.0 (GraphPad Software), accounting for ligand depletion. All numerical data are presented as mean ± SEM for at least three independent experiments.

### Synthesis of α-Ctx RgIA Globular Isomer

The globular isomer of α-Ctx RgIA was synthesized similarly to Ellison et al. (2008) by the solid-phase method, using Fmoc-protected amino acids with the Trt- and Acm- protection of cysteines for the first (1–3) and second (2–4) disulfide bonds, respectively, and DIC/HOBt as a coupling reagent. Linear peptide was totally deprotected and cleaved from the polymer with TFA/DTT/H2O 93:4:3 cocktail. A crude peptide was isolated by ether precipitation and subsequent purification was performed on YMC Triart C18 10 u 150 mm × 30 mm column, using Gilson 333/334 binary gradient HPLC system with spectrophotometric detection at 210-nm wavelength. The purified linear RgIA was subjected to a closure of the first disulfide bond by atmospheric oxygen at high pH. Briefly, linear peptide was dissolved in aqueous 50 mM ammonium bicarbonate to a final concentration of 0.5 g/L, stirred on air for 72 h and lyophilized. Purification of monocyclic intermediate was performed under the same conditions as linear ones. Deprotection of Acm-protected cysteines and oxidation were performed by treatment with iodine solution in glacial acetic acid. Excess of iodine was quenched by aqueous citric acid. The reaction mixture was freeze-dried, and the final product was purified on a C8 reverse-phase column. Its purity was confirmed by UPLC/MS analysis.

### Crystallization and Data Collection

Crystals of the deglycosylated α9-ECD in complex with α-Ctx RgIA were grown by the sitting-drop vapor-diffusion method in 100 mM HEPES, pH 7.5, 20% PEG 10000 at a protein concentration of 3.5 mg ml^−1^ and a molar ratio of protein to RgIA equal to 1:3. Cryoprotection of the crystals was achieved by immersion in a solution containing the precipitant and 20% ethylene glycol for ∼10 s. Subsequently, the crystals were vitrified in liquid N_2_. Data were collected at 100 K at a wavelength of 1.0 Å on beamline I04 of the Diamond Light Source, Didcot, United Kingdom. The reflections were integrated with XDS (Kabsch, 2010), the space group was determined with POINTLESS (Evans, 2011), and the data merging was carried out with SCALA (Evans, 2006) of the CCP4 (Winn et al., 2011) suite.

### Structure Determination and Refinement

The structure of the α9-ECD/RgIA complex was solved by molecular replacement with PHASER (McCoy et al., 2007), using as a search model the apo structure of α9-ECD (PDB ID: 4D01) (Zouridakis et al., 2014). The electron density maps clearly showed the presence of a large and continuous electron density in the binding site region, attributed to α-Ctx RgIA. The structure was refined with PHENIX (Afonine et al., 2012) with restrained refinement and TLS refinement implemented in the final stages. Model building and real-space refinement were performed in COOT (Emsley et al., 2010). The high-resolution limit was determined with the CC1/2 and mean (I/σI) criteria (Karplus and Diederichs, 2012), using as cutoff the values of 60% and 1.5, respectively. Almost 99% of the residues of α9 ECD were in Ramachandran favored or allowed regions, and 1% were outliers, whereas the overall geometry inspection showed no outliers in rotameric and omega angle analyses. The electron density for the region 102–104 of α9-ECD could not be determined and therefore the corresponding residues were not built in the model. The atomic coordinates and structure factors of the α9-ECD/RgIA complex were deposited in the Protein Data Bank, under the accession code 6HY7. The PyMOL program^1^ was used for structure visualization and for generation of the figures.

### Computational Methods

The homology model of human nAChR α10-ECD was based on the X-ray crystal structure of human α9-ECD complex with the α-Ctx RgIA presented here (PDB ID: 6HY7). All non-protein atoms were removed from the template structure. Sequence alignment between human α9 and α10 ECDs (Figure 2D) was performed using Clustal Omega and the UNIPROT accession codes Q9UGM1 and Q9GZZ6 for α9 and α10, respectively (67% sequence identity for 212 residues). From a total of 30 homology models of human α10-ECD that were generated using Modeller v9.10 (Fiser and Sali, 2003), we selected the lowest DOPE score model, which was used without any further optimization. The models of pentameric (α9)_2_(α10)_3_ and (α9)_3_(α10)_2_ ECDs were prepared by superimposing the two human monomers (either free or RgIA-bound) on the crystallographic structure of the *Aplysia californica* AChBP in complex with α-Ctx ImI (PDB ID: 2C9T) (Ulens et al., 2006), using the MULTISEQ plugin of VMD v1.9.4 (Humphrey et al., 1996). RgIA was placed at only one binding site in the modeled pentamers, formed between either α9(+)/α9(−), or α9(+)/α10(−), or between α10(+)/α9(−) interfaces.

### Molecular Dynamics (MD) Simulations

Molecular dynamics simulations were performed using the GPU-accelerated version of PMEMD in AMBER v16 (Case et al., 2005; Salomon-Ferrer et al., 2013) and the ff14SB force field parameters (Maier et al., 2015). The systems were solvated in truncated octahedron boxes of TIP3P waters with a minimum extension of 12 Å from the solute and the total charge was neutralized with the addition of sodium ions. All simulations were performed with a 4-fs time step by applying the hydrogen mass repartitioning method (Hopkins et al., 2015). The Particle Mesh Ewald method was used for long-range electrostatic interactions with a real space cutoff of 9 Å. Temperature was regulated using a Langevin thermostat with a collision frequency of 1.0 ps^−1^ and the pressure was regulated at 1.0 bar using the Berendsen weak-coupling algorithm with a relaxation time of 1.0 ps. First, each system was energy minimized without restraints for 2000 steps to remove steric clashes. Then, the temperature was increased to 200 K within 100 ps under constant volume (NVT ensemble), using harmonic positional restraints of 100 kcal/mol Å^2^ on all protein atoms. Within the next 200 ps, temperature was increased to 300 K under constant pressure (NPT ensemble), while reducing the restraints to 50 kcal/mol Å^2^. Pressure equilibration was performed for a total of 2 ns at 300 K in the NPT with restraints applied only on the (+) side and RgIA Cα atoms. These restraints were gradually decreased from 25 to 1.0 kcal/mol Å^2^ within the first 1 ns of equilibration and were retained during the remaining equilibration. In subsequent unrestraint production simulations of 0.5 μs at 300 K in the canonical ensemble (NVT) we observed that RgIA sampled a large conformational space rendering analysis of residue-specific interactions at either (+) or (−) sides very difficult. Therefore, we retained weak restraints of 1.0 kcal/mol Å^2^ only on the 211 Cα atoms of the α9 or α10 ECD comprising each time the (+) side of the binding site and on the 13 Cα atoms of RgIA for 0.2 μs of the production runs. These restraints were then reduced to 0.1 kcal/mol Å^2^ for additional 0.2 μs, while the side chains of all residues were kept unrestrained during the whole simulation time. In contrast, the ECD participating at the (−) side of the binding site and the other three ECDs in each pentameric system were unrestrained, allowing for the sampling of potential interactions of RgIA with the (−) side of the α9 or α10 ECD. Trajectory snapshots were collected every 10 ps and were processed using the CPPTRAJ module of AMBER. The non-bonded interaction energy terms (electrostatic and van der Waals) between RgIA and the nAChR ECDs were calculated within the LIE methodology implemented in CPPTRAJ with the default 12-Å cutoff. Clustering of the trajectory snapshots was performed using a hierarchical agglomerative approach with a minimum distance between clusters of 2.0 Å, after mass-weighted, root-mean-square deviation fitting of the Ca atoms at the two binding subunits of RgIA. Calculations were performed on Linux workstations equipped with NVIDIA GTX 1080 GPUs.

## Results

### Overall Structure of the α9-ECD/RgIA Complex

**FIGURE 1:**
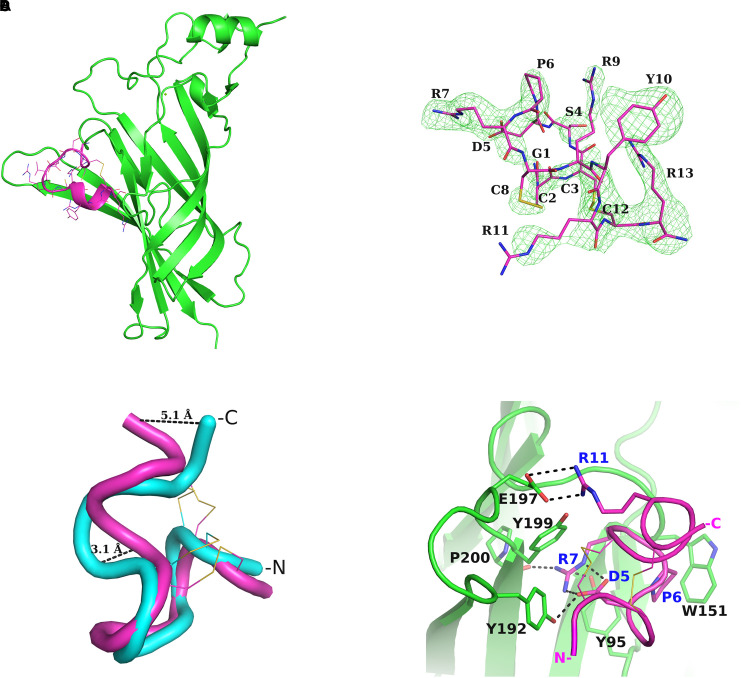
Structure of α-Ctx RgIA bound to nAChR α9-ECD and interactions. **(A)** Overall structure of the complex of monomeric α9-ECD (in green) with α-Ctx RgIA (in magenta). **(B)** Simulated annealing omit map of the region of the bound RgIA, contoured at 3σ. **(C)** Superposition of the NMR structure of RgIA (in cyan) with its crystal structure (in magenta) when bound to α9-ECD, determined in the present study. The highly diverging regions, at the middle helical domain and at the C-terminus, are shown. **(D)** Interaction scheme of RgIA with the (+) side of α9-ECD. The interacting residues are drawn in sticks and the polar or charged interactions are shown in dashed lines. Blue labels account for the RgIA-interacting residues and black labels for the α9-ECD-interacting residues.

The crystal structure of the complex of the deglycosylated human nAChR α9-ECD in complex with the C-terminally amidated α-Ctx RgIA was solved at 2.26-Å resolution (Figure 1A,B, Supplementary Figure S1 and Supplementary Table S1). Whereas the structure of the α9-ECD in this complex was very similar to the previously determined structure of the apo α9-ECD (Zouridakis et al., 2014), presenting a RMSD value of 0.497 Å for their paired Cα atoms, the crystal structure of RgIA bound to α9-ECD presented an RMSD value for Cα atoms of 1.905 Å compared to its NMR structure in solution (PDB ID: 2JUT) (Ellison et al., 2008). This difference is mainly attributed to the C-terminal Arg13, which upon superposition of the two RgIA structures shows >5 Å distance between their Cα atoms, revealing the intrinsic flexibility of this residue (Figure 1C). Another notable difference is the α-helical domain in the middle of the crystallized RgIA molecule, which is missing in the NMR structure of RgIA (Figure 1C). In addition, in the crystal structure of RgIA, an intramolecular salt bridge between Asp5 and Arg7 was observed (Figure 1D), also missing from its NMR structure.

**FIGURE 2:**
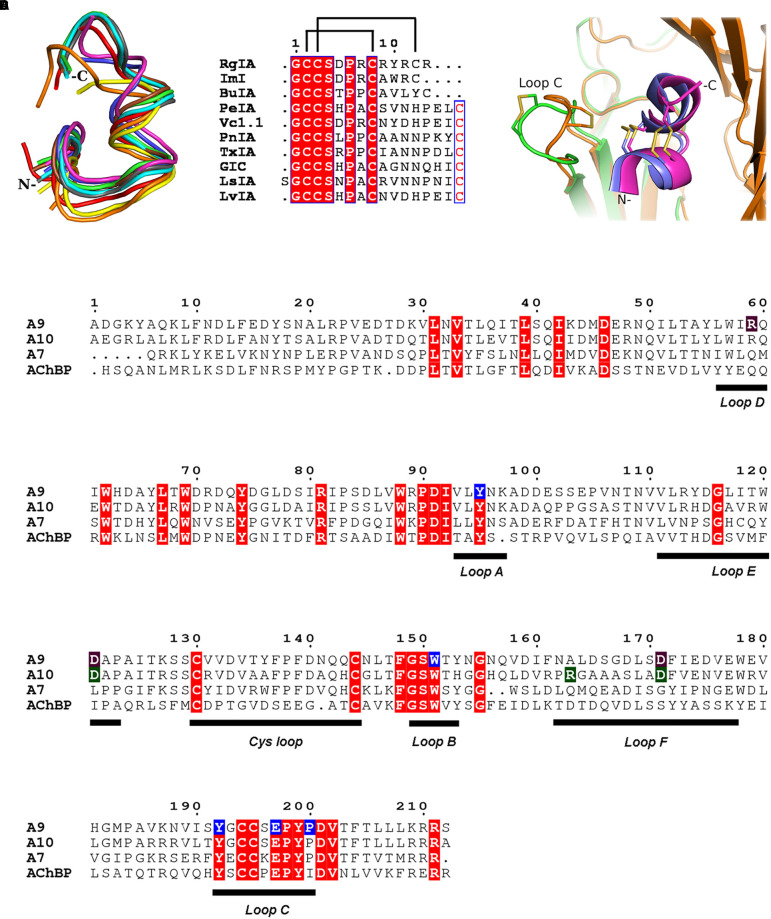
α-Ctxs bound to α9-ECD and homologous proteins. **(A)** Overlay of all crystallized α-Ctxs bound to AChBPs and of RgIA bound to α9-ECD, as a result of the superposition of the protomers of AChBPs to the monomeric α9-ECD (RgIA in magenta, ImI in blue, PeIA in cyan, GIC in green, PnIA in gray, LsIA in red, LvIA in brown, BuIA in yellow, and TxIA in orange). **(B)** Sequence alignment of crystallized α-Ctxs with AChBPs or α9-ECD. Disulfide bridges in RgIA are indicated by lines on top of the alignment. **(C)** Comparison between the AChBP/ImI and α9-ECD/RgIA complexes. AChBP in orange, α9-ECD in green, ImI in blue, and RgIA in magenta. **(D)** Sequence alignment of human α9, α10, α7 nAChR-ECDs and *Lymnaea stagnalis* AChBP. α9(+) side-interacting residues with RgIA, as shown in the crystal structure, in blue boxes; α9(–) and α10(–) interacting residues with RgIA, as shown in the MD studies, in purple and green, respectively.

To investigate whether these observed conformational changes of RgIA upon binding to α9-ECD have been reported in other cases, we sought for examples involving α-Ctxs. When comparing the NMR structures of PnIA (PDB ID: 1PEN) (Hu et al., 1996), GIC (PDB ID: 1UL2) (Chi et al., 2004), BuIA (PDB ID: 2I28) (Chi et al., 2006) and LvIA (PDB ID: 2MDQ) (Luo et al., 2014) to their crystal complexes with AChBP (PDB IDs: 2BR8 for PnIA; 5CO5 for GIC; 4EZ1 for BuIA; 5XGL for LvIA) (Celie et al., 2005; Lin et al., 2016; Xu et al., 2017), no differences were noticed regarding their backbone conformations. However, in the case of ImI, which is the most similar α-Ctx to RgIA (Figure 2B), two clusters of NMR structures are available; one lacking the α-helical domain in the middle of the toxin (PDB ID: 1CNL) (Gehrmann et al., 1999), similarly to the sole NMR structure of RgIA (PDB ID: 2JUT) (Ellison et al., 2008), and another where the helical domain is present (PDB ID: 1IMI) (Maslennikov et al., 1999) (Supplementary Figure S2), as in its crystal complex with AChBP (PDB ID: 2C9T) (Ulens et al., 2006). Thus, it is not clear whether the observed backbone conformational differences between the crystallized RgIA bound to α9-ECD and its NMR structure are induced by its interactions with α9-ECD or could be due to varying in-solution NMR conformational states (as in the case of ImI). Instead, the salt bridge between Asp5 and Arg7 of RgIA is most probably induced by its binding to α9-ECD, since this is also apparent in the crystal complex of ImI with AChBP, while lacking from its varying NMR structures (Supplementary Figure S2).

Comparing the α9-ECD/RgIA complex with the structures of AChBPs in their complexes with other α-Ctxs, a strikingly high structural similarity was observed for the bound toxins (Figure 2A), despite their differences in sequence composition and length (Figure 2B). Specifically, upon superposition of the α9-ECD bound to RgIA with the protomers of AChBPs bound to other α-Ctxs, it was revealed that RgIA has an RMSD value for all paired Cα atoms of 0.773 Å with PnIA (PDB ID: 2BR8) (Celie et al., 2005), 0.778 Å with PeIA (PDB ID: 5JME) (Hone et al., 2018), 0.702 Å with ImI (PDB ID: 2C9T) (Ulens et al., 2006), 0.865 Å with TxIA (PDB ID: 2UZ6) (Dutertre et al., 2007), 1.013 Å with BuIA (PDB ID: 4EZ1), 1.059 Å with GIC (PDB ID: 5CO5) (Lin et al., 2016) and of 1.321 or 0.979 Å with LsIA (PDB ID: 5T90) (Abraham et al., 2017) or LvIA (PDB ID: 5XGL) (Xu et al., 2017), respectively.

Thus, all α-Ctxs in the above complexes adopt similar orientations, but they may be further grouped into three distinct clusters, regarding their backbone trajectories in the above complexes. The α4/3-Ctxs ImI and RgIA comprise the first group, the α4/7-Ctxs GIC, LsIA, LvIA, PeIA, and PnIA fall into the second group and the α4/4-Ctx BuIA is grouped together with the α4/7-Ctx TxIA (Supplementary Figure S3). The members of the first and second groups present an apparent spatial coincidence up to their ninth residue, with RgIA and ImI deviating beyond this point from the other toxins, which on the other hand form a two-turn extended α-helical domain (Supplementary Figure S3). RgIA and ImI are identical up to their Cys8 residue and differ in two residues at positions 9 and 10 (ImI-Ala9 and ImI-Trp10 vs. RgIA-Arg9 and RgIA-Tyr10) with RgIA being longer than ImI by a C-terminal Arg residue (Figure 2B).

Taken the above into consideration, despite α9-ECD being in a monomeric state, it binds α-Ctx-RgIA in a similar fashion to that of binding of other α-Ctxs to the pentameric AChBPs (Figure 2A), as previously shown for the complexes of α9-ECD with α-Bgtx and MLA (Zouridakis et al., 2014). This becomes more evident in Figure 2C, which shows the overall very good superposition between the complexes of α9-ECD with RgIA and of AChBP with ImI (Ulens et al., 2006). The above observations suggest that the resolved structure presented here depicts accurately the orientation of RgIA in the binding sites of pentameric α9α10 nAChRs where α9 contributes its (+) side. It should be mentioned that the crystal packing contacts between the bound RgIA and an adjacent symmetric α9-ECD, contributing to the stabilization of the α9-ECD/RgIA complex, affected only the orientation of the side chain of RgIA-Tyr10, as revealed after comparison to its counterpart ImI-Trp10 in the crystal structure of ImI bound to AChBP (Supplementary Figure S4).

### Interactions of RgIA With α9-ECD

Upon binding, RgIA is buried in the (+) side of α9-ECD (Figure 1A) and shares a common orientation with other previously determined α-Ctxs bound to AChBPs (Figure 2A,C). Its central helical domain protrudes toward the binding site, while its N- and C-termini are located at the bottom and top of the binding site, respectively (Figure 1A, 2C).

The most profound interactions of RgIA with the (+) side of α9-ECD involve its aspartic residue at position 5 and its arginine residues at positions 7 and 11 (Figure 1D, 2B). RgIA-Asp5 forms a H-bond with loop-C α9-Tyr192, RgIA-Arg7 forms H-bonds with the carbonyl oxygen of loop-C Pro200 and the hydroxyl group of loop-A Tyr95, while RgIA-Arg11 makes a salt bridge with loop-C Glu197 (Figure 1D). Additionally, RgIA-Pro6 makes van der Waals interactions with the loop-B Trp151. Notably, the similar α4/3-Ctx ImI makes identical interactions with the (+) side of the binding site of AChBP (Hansen et al., 2005; Ulens et al., 2006), which nevertheless involve highly conserved residues among AChBPs and nAChR α subunits.

### Radio-Ligand Competition Experiments

**FIGURE 3:**
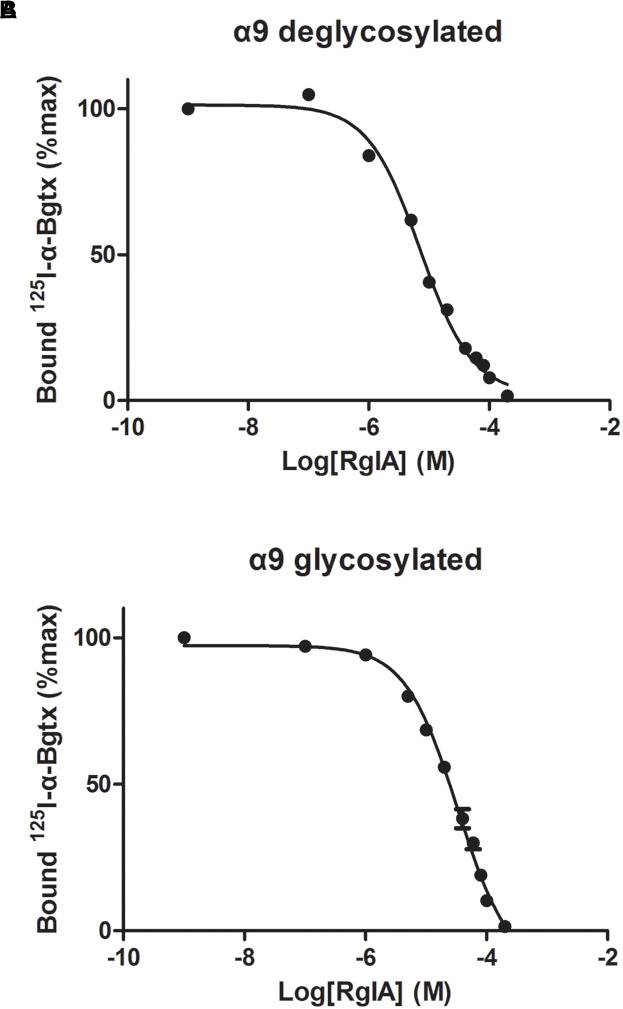
Competition of radiolabeled α-Bgtx by RgIA. **(A)** On deglycosylated α9-ECD **(B)** On glycosylated α9-ECD. Each graph is from four experiments and standard errors are presented with bars.

The binding affinity of α-Ctx RgIA to α9-ECD was determined via competition experiments, using the monomeric glycosylated or deglycosylated α9-ECD and radiolabeled [^125^I]-α-Bgtx (Figure 3). The K_i_ values for the two α9-ECDs, differing in their glycosylation state, were slightly different, with the deglycosylated α9-ECD (the one co-crystallized with RgIA) having 2.7 ± 0.3 μM, and the glycosylated one having 13.3 ± 1.5 μM. Therefore, one could suggest that the interactions presented in the obtained crystal structure with the deglycosylated α9-ECD, depict those occurring in the case of the glycosylated native α9α10 nAChRs, concerning the α9(+) side. Also, these K_i_ values are in the low micromolar range, as the calculated IC_50_ values for RgIA in human α9α10 nAChRs (Azam and McIntosh, 2012).

### Molecular Modeling and MD Simulations of Pentameric Human α9α10 ECDs

**FIGURE 4:**
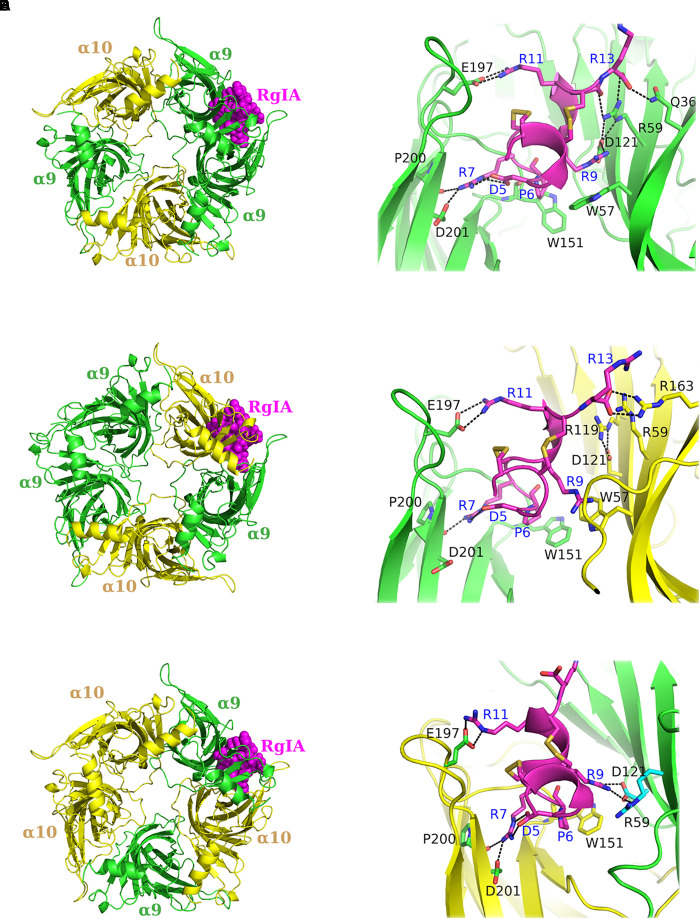
Molecular dynamics (MD) models of α9α10 nAChR-ECDs. **(A,B)** RgIA bound to the α9(+)/α9(−) interface and close view of interactions. **(C,D)** RgIA bound to the α9(+)/α10(−) interface and close view of interactions. **(E,F)** RgIA bound to the α10(+)/α9(−) interface and close view of interactions. α9-ECD in green, α10-ECD in yellow and RgIA in magenta. Labeled in blue are the RgIA-interacting residues and in black the interacting residues of α9 or α10-ECDs.

With the aim to gain more information about the potential interactions between α-Ctx RgIA and the ligand-binding sites of human α9α10 nAChR, we modeled both possible stoichiometries of the α9α10 nAChR ECD (Indurthi et al., 2014), namely (α9)_2_(α10)_3_ and (α9)_3_(α10)_2_ (Figure 4), based on the crystal structures of the complexes α9-ECD/RgIA (Figure 1A) and AChBP/ImI (Ulens et al., 2006). In the case of the (α9)_3_(α10)_2_, an additional binding site is formed between α9 ECDs (Indurthi et al., 2014), apart from that between α9 and α10 ECDs in the (α9)_2_(α10)_3_ stoichiometry (Plazas et al., 2005). One RgIA molecule was modeled in the α9(+)/α9(−) or in the α9(+)/α10(−) binding site in the (α9)_3_(α10)_2_ ECD model (Figure 4A,C), or in the α10(+)/α9(−) binding site in the model of (α9)_2_(α10)_3_ ECD (Figure 4E) (all constructed models are provided as Supplementary Material in pdb format). Subsequently, we employed MD simulations for the modeled complexes.

**FIGURE 5:**
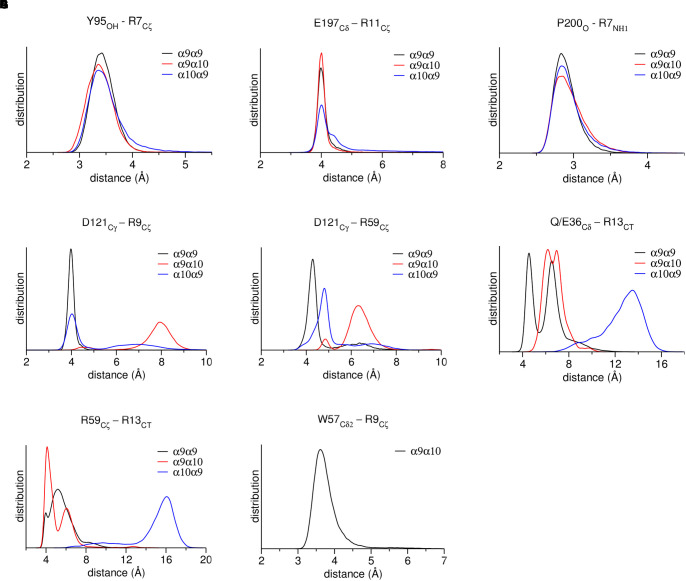
Distribution of distances between characteristic residues, throughout MD simulations. **(A)** α9 or α10 loop-A Tyr95 with RgIA-Arg9. **(B)** α9 or α10 loop-C Glu197 with RgIA-Arg11. **(C)** α9 or α10 loop-C Pro200 with RgIA-Arg7. **(D)** α9 or α10 loop-E Asp121 with RgIA-Arg9. **(E)** Loop-E Asp121 with loop-D Arg59 in α9 or α10 subunits. **(F)** α9-Gln36 or α10-Glu36 with RgIA-Arg13. **(G)** α9 loop-D Arg59 with RgIA-Arg13. **(H)** α10 loop-D Trp57 with RgIA-Arg9.

Our MDs revealed that the interactions of RgIA at the (+) sides of the binding sites conferred by α9 or α10 ECDs were almost identical, given the high sequence similarity (77%) between these ECDs (Figure 2D). More specifically, the conserved residues Tyr95 and Glu197 formed a hydrogen bond and a salt bridge with RgIA Arg7 and Arg11, respectively, while the conserved Pro200 interacted with a strong hydrogen bond with RgIA-Arg7 (Figure 4B,D,F). These interactions were retained throughout the MD simulations (Figure 5A–C) and were essentially identical to those shown in the crystal structure of α9-ECD with RgIA (Figure 1D). In addition, similarly to the crystal structure, RgIA-Pro6 interacts favorably with the loop-B Trp151 of both α9(+) and α10(+) sides via CH_2_**-**π interactions (Figure 4B,D,F).

At the α9(−) side of either α9(+)/α9(−) or α10(+)/α9(−) binding sites (Figure 4A,E), Asp121 and RgIA-Arg9 formed a salt bridge (Figure 4B,F), which was stable throughout the course of MD simulations (Figure 5D). At both binding sites, α9-Asp121 formed a stable intramolecular interaction with α9-Arg59 (Figure 4B,F, 5E), an interaction present in all previous α9-ECD crystal structures (Zouridakis et al., 2014), as well as in the α9-ECD/RgIA structure, presented here. In the α9(+)/α9(−) binding site, distinctly to the α10(+)/α9(−) binding site, the MD simulations revealed interactions between α9-Gln36 and α9-Arg59 with the carboxylate group of the RgIA C-terminus (Figure 4B), which however were short-lived (Figure 5F,G), probably due to the high mobility of the RgIA C-terminus, and thus should be considered only transient.

At the α10(−) side of the α9(+)/α10(−) binding site (Figure 4C,D), a complete different motif of interactions with RgIA was revealed, probably due to the high accumulation of arginine residues at the α10(−) side (Figure 4D). Interestingly, α10-ECD bears two additional arginine residues at positions 119 and 163 of its −) side compared to α9-ECD (Figure 2D, 4D). The stable interaction observed between Asp121 and RgIA-Arg9, when α9 is involved in the (−) side, is no longer present, nor is the intramolecular bond between Asp121 and Arg59 (Figure 4D, 5D,E). Instead, other intramolecular interactions, such as those between Asp121 and Arg119 or between Arg59 and Glu61, stabilize the α10(−) side, but its interactions with RgIA are rather aberrant and transient, apart from the cation-π interaction between α10-Trp57 and RgIA-Arg9 (Figure 4D, 5H).

## Discussion

In this study, we determined the crystal structure of the complex of human neuronal nAChR α9-ECD with α-Ctx RgIA, revealing the interactions of RgIA with the α9(+) side (Figure 1A,D). The interaction motif of RgIA with α9-ECD is identical to that of α-Ctx ImI with AChBP (Hansen et al., 2005; Ulens et al., 2006), since the involved residues of the two α-Ctxs are identical and the interacting residues of both proteins are highly conserved. Both α-Ctxs interact with α9(+) or AChBP(+) sides through their Asp-Pro-Arg triad of loop 1 and a conserved Arg at position 11 (Figure 2B). These observations comply with previous mutational studies, which had shown the critical role of the Asp-Pro-Arg triad to the inhibitory potency of RgIA on α9α10 nAChRs (Ellison et al., 2006, 2008).

Moreover, since the complex of the monomeric α9-ECD with RgIA superimposed very well with the complexes of the pentameric AChBPs with ImI (Figure 2C) and other α-Ctxs (Figure 2A), this study showed that the (+) side of the α9-ECD alone is adequate to determine the orientation of the bound RgIA. This is similar to previous observations for the complexes of the monomeric α9-ECD with the antagonists MLA and α-Bgtx (Zouridakis et al., 2014). The K_i_ value of RgIA binding to the deglycosylated α9-ECD was calculated to be 2.7 μM, similar to that for the glycosylated protein (13 μM), which is the form more close to the native α9 subunit (Figure 3). Taking also into account that the IC_50_ value of RgIA to human α9α10 nAChRs is also at the low micromolar range (∼0.5 μM) (Azam and McIntosh, 2012), the interactions revealed in the structure of the deglycosylated α9-ECD with RgIA are very likely to depict the ones occurring in the α9(+) side-containing binding sites of native human α9α10 nAChRs.

Notably, α9 and α10 ECDs have a remarkable sequence similarity of 77%, which is even higher for the regions participating in the (+) side (loops A, B, and C) of the binding site (Figure 2D). In particular, the (+) sides of the two ECDs differ only in one residue at position 153; α10 has a histidine residue in contrast to tyrosine in α9 and all other nAChR α-subunits. However, this highly conserved tyrosine, as shown in the crystal structures of α9-ECD and other homologous proteins (e.g., Brejc et al., 2001; Kouvatsos et al., 2016; Morales-Perez et al., 2016), faces toward the interior of the protein and has not been considered a binding determinant in nAChRs (Hansen et al., 2005; Ulens et al., 2006). Thus, since the α9(+) and α10(+) sides are almost identical, one can expect that the α10(+) side could also bind RgIA similarly.

Given that α9α10 nAChRs may contain three putative binding sites, namely the α9(+)/α9(−), α9(+)/α10(−), and the α10(+)/α9(−) (Plazas et al., 2005; Ellison et al., 2008; Azam and McIntosh, 2012; Indurthi et al., 2014; Azam et al., 2015; Boffi et al., 2017), we performed MD simulations in order to assess possible preference of RgIA for any of these sites, conferred by the non-conserved (−) sides of α9 or α10 ECDs. Previous attempts of modeling the complex of RgIA with the ECD of α9α10 nAChR have yielded controversial results: Perez et al. (2009) suggested that the favorable binding site for RgIA is the α9(+)/α10(−), whereas Azam et al. (2015) proposed the α10(+)/α9(−), complying with their mutational and electrophysiological data. However, these models were based on the X-ray crystal structures of either the AChBP/ImI complex alone (Ulens et al., 2006), or on the α9-ECD apo structure (Zouridakis et al., 2014) and AChBP/ImI complex. In the current study, the X-ray structure of α9-ECD in complex with RgIA was used as a template together with the AChBP/ImI complex for modeling of the binding sites of the human α9α10 nAChR.

As expected, due to the substantial similarity of the (+) sides of α9 and α10 ECDs, the MD studies showed that RgIA forms similar interactions with them (Figure 4B,D,F), some of which have been previously evaluated by mutational studies. The mutation α9-Trp151 to threonine has led to a ∼10-fold increase of the IC_50_ value of RgIA to α9α10 nAChRs (Ellison et al., 2008), while the single-point mutations Glu197Gln or Pro200Gln at the α10(+) side have shown a ∼20- or ∼400-fold decrease in the potency of RgIA to α9α10 nAChRs (Azam et al., 2015).

**FIGURE 6:**
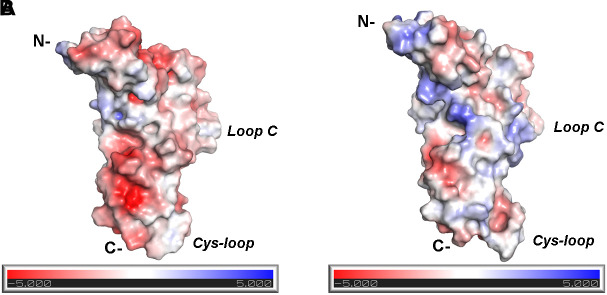
Electrostatic potential distribution at the (–) sides of α9 and α10 nAChR-ECDs. **(A)** α9-ECD. **(B)** α10-ECD.

Instead, the interactions formed between RgIA and the non-conserved (−) sides of α9 or α10 ECDs are significantly different:

(a) At the α9(+)/α9(−) and α10(+)/α9(−) interfaces, the (−) side of α9-ECD forms a critical salt bridge between α9-Asp121 and RgIA-Arg9 (Figure 4B,F, 5D). This interaction was previously shown to be very important for the potency of both ACh and RgIA on α9α10 nAChRs, since mutation α9-Asp121Leu increased the corresponding EC_50_ or IC_50_ values by ∼30 times or by three orders of magnitude, respectively (Azam et al., 2015).

(b) At the α9(+)/α10(−) interface, where the (−) side of α10-ECD is densely populated by positively charged residues (Figure 4D, 6), the interaction of Asp121 with RgIA-Arg9, shown at the α9(+)/α9(−) and α10(+)/α9(−) interfaces, is disrupted; instead, α10-Asp121 makes an intramolecular salt bridge with α10-Arg119, whereas RgIA-Arg9 makes a cation-π interaction with the α10-Trp57 (Figure 4D). However, this interaction in α9(+)/α10(−) interfaces, if applicable in nature, could not justify the selectivity of RgIA on α9α10 nAChRs, since this loop-D tryptophan residue is invariant among all nAChR subunits. The observations for the modeled α9(+)/α10(−) interface comply with previous functional data which showed that the mutation α10-Asp121Leu did not affect the inhibitory potency of RgIA on α9α10 nAChRs (Azam et al., 2015).

The differences in the binding motif of RgIA with the α10(−) side compared to the α9(−) side may be attributed to repulsive forces between the profoundly more positively charged α10(−) side (Figure 6) and the positively charged RgIA. A first indication supporting the role of electrostatics in the binding of RgIA is the calculated non-bonded interaction energies extracted from the MD simulations of each pentameric assembly. The electrostatic term of the interaction energy (*E*_elec_) was calculated to be −743 ± 37 kcal/mol for α9(+)α9(−), −620 ± 87 kcal/mol for α10(+)α9(−), and −498 ± 86 kcal/mol for α9(−)α10(+). The corresponding van der Waals interaction energy terms (*E*_vdW_) are calculated to be −95 ± 11 kcal/mol α9(+)α9(−), −74 ± 10 kcal/mol for α10(+)α9(−), and −69 ± 17 kcal/mol for α9(+)α10(−). In addition, the interface areas formed by two adjacent subunits with bound RgIA, in the cases of α9(+)/α9(−) and α10(+)/α9(−) are 1084 ± 121 Å^2^ and 1050 ± 80 Å^2^, respectively, close to the experimentally determined one (1194 ± 33 Å^2^) from the structure of AChBP with α-Ctx ImI (Ulens et al., 2006). Instead, in the case of α9(+)/α10(−), the interface area is significantly lower (685 ± 79 Å^2^), indicating a rather aberrant assembly between these two subunits in the presence of α-Ctx RgIA. Taken together, our results indicate the lower affinity of RgIA binding at the α9(+)α10(−) site with respect to the α9(+)α9(−) and α10(+)α9(−), in well agreement with previous mutational and functional data, which have shown that the (+) side of RgIA binding is conferred by either α9 or α10 subunits (Ellison et al., 2008; Azam et al., 2015) and the (−) side by α9 rather than α10 (Azam and McIntosh, 2012; Azam et al., 2015). It seems plausible that the arginine residues at positions 119 and 163 at the (−) side of α10-ECD, which in the case of α9 correspond to threonine and alanine, respectively (Figure 2D), contribute critically to the repulsion of α-Ctx RgIA in α10(−) side-containing binding sites. It is noteworthy that α10 is the only nAChR α-subunit bearing charged residues at these sites.

In the presented crystal structure of the complex of α9-ECD with RgIA, and in the structures of the free α9-ECD and of its complexes with α-Bgtx and MLA (Zouridakis et al., 2014), Asp121 forms a stable salt bridge with the adjacent Arg59. Notably, these charged residues are uniquely present in the α9 and α10 nAChR subunits. Our MD simulations showed that this interaction was retained in the α9(−) side in both α9(+)/α9(−) and α10(+)/α9(−) binding sites, but was disrupted in the α10(−) side throughout the simulations (Figure 5E), despite its presence in the initial α10-ECD model. Thus, in the case of the α9(+)/α10(−) binding site, where the (−) side of α10-ECD is more positively charged, structural rearrangements of residues at the α10(−) side occurred in the course of MD simulations, in order to accommodate the also positively charged RgIA, leading to different interactions with RgIA (Figure 4D) compared to those with the α9(−)side (Figure 4B,F).

Interestingly, other α9α10 selective α-Ctxs are PeIA and Vc1.1, which despite being α4/7-Ctxs, have a potency for the rat α9α10 nAChR (IC_50_ of 7 or 19 nM, respectively) comparable with that of RgIA (IC_50_ = 4.5 nM) (McIntosh et al., 2005; Clark et al., 2006; Vincler et al., 2006). PeIA and Vc1.1 have almost identical loop-2 compositions, but completely different than that of RgIA (Figure 2B). In addition, PeIA lacks the loop-1 Asp-Pro-Arg triad, shown to make critical interactions with the α9(+) side in the α9-ECD/RgIA crystal structure and with the (+) sides of the presented α9α10 nAChR-ECD models. Thus, the selectivity of these α-Ctxs to α9α10 nAChRs has to deal with other interactions than those between RgIA and the receptor.

Several models of pain and inflammation have demonstrated that α9α10 nAChRs play a role in modulating the pathophysiology associated with neuropathic pain (Di Cesare Mannelli et al., 2014; Pacini et al., 2016). The analgesic and anti-inflammatory effects of RgIA and Vc1.1 via inhibition of the α9α10 nAChR have also been demonstrated (Satkunanathan et al., 2005; Vincler et al., 2006). However, it has been proposed that the effects of these α-Ctxs can also be transmitted via inhibition of GABA_β_ receptors (Callaghan et al., 2008; Sadeghi et al., 2017). Vc1.1 was tested as the first nAChR-targeting α-Ctx for the treatment of neuropathic pain, but since it was demonstrated that, similarly to RgIA (Azam and McIntosh, 2012), Vc1.1 was several orders of magnitude less potent in humans than in rats (Terlau and Olivera, 2004; Halai et al., 2009), clinical trials were discontinued. The difference in potency of RgIA between human and rat α9α10 nAChRs has been attributed to a specific residue located at position 61 of α9 (Ile in humans vs. Thr in rats), at its (−) side (Azam and McIntosh, 2012). Previous MD studies indicated that this threonine residue in rat α9α10 nAChRs coordinates a network of interactions within α9 (−) side, facilitating the binding of RgIA to these receptors (Azam et al., 2015). Efforts to improve the potency of RgIA in human α9α10 nAChRs have led to an over 1000-fold more potent analog (RgIA4) (Romero et al., 2017), shown to be an effective analgesic in a model of neuropathic pain (Christensen et al., 2017; Romero et al., 2017), while being highly selective for α9α10 nAChRs over GABA_β_ receptors. Notably, in this analog, among other drastic changes, the positively charged Arg residues at positions 9, 11, and 13 were replaced by the neutral citrulline, Qln, and Tyr residues, respectively. These replacements have probably alleviated the repulsive forces between RgIA and (−) sides of α9α10 nAChRs, while maintaining the ability of RgIA to make interactions with α9α10 nAChRs, but via H-bonding. However, in order to deeply understand the interactions of RgIA4 with α9α10 nAChRs, additional detailed structural studies involving RgIA4 are needed. The findings of the present study, showing the actual interactions of RgIA with the (+) side of the human α9-ECD and the indicative interactions in the fully assembled binding sites of the human α9α10 nAChR ECD, may be helpful for the design of improved therapeutic analogs.

## Conclusion

The first crystal structure of a human nAChR domain with an α-Ctx is presented. The structure revealed the interactions between α-Ctx RgIA and the (+) side of neuronal nAChR α9-ECD in high detail. Based on the structure of this complex, models of human α9α10 nAChR ECD with fully formed binding sites were constructed with RgIA bound to each of them. Our MD simulations suggest that the favorable binding site of RgIA in the human α9α10 nAChR ECD consists of either α9 or α10 subunits as the (+) side and of an adjacent α9 rather than α10 subunit as the (−) side. The results of this study may be helpful to medicinal chemists for design of improved RgIA analogs targeting the human α9α10 nAChR against auditory diseases and neuropathic pain.

## Author Contributions

MZ and PG conceived the project, expressed and purified α9-ECD, conducted the competition experiments, crystallized the complex of α9-ECD with α-Ctx RgIA and solved the crystal structure. PG, MZ, and IK designed the experiments. II and IK synthesized and purified the α-Ctx RgIA and AP performed and analyzed the MD simulations. PG, MZ, and AP wrote the manuscript. ST and VT contributed to management of the project and edited the manuscript. All authors reviewed and approved the manuscript.

## Conflict of Interest Statement

The authors declare that the research was conducted in the absence of any commercial or financial relationships that could be construed as a potential conflict of interest.

## Footnotes

1
http://www.pymol.org/

## Abbreviations

ACh: acetylcholine
AChBP: acetylcholine-binding protein
ECD: extracellular domain
MD: molecular dynamics
MLA: methyllylcaconitine
nAChRs: nicotinic acetylcholine receptors
RMSD: root mean square deviation
α-Bgtx: α-bungarotoxin
α-Ctx: α-conotoxin
(+) side: principal side
(−) side: complementary side.
