# Corrigendum: Crystal structure of the monomeric extracellular domain of α9 nicotinic receptor subunit in complex with α-conotoxin RgIA: Molecular dynamics insights into RgIA binding to α9α10 nicotinic receptors

**DOI:** 10.3389/fphar.2022.980202

**Published:** 2022-08-25

**Authors:** Marios Zouridakis, Athanasios Papakyriakou, Igor A. Ivanov, Igor E. Kasheverov, Victor Tsetlin, Socrates Tzartos, Petros Giastas

**Affiliations:** ^1^ Department of Neurobiology, Hellenic Pasteur Institute, Athens, Greece; ^2^ Institute of Biosciences and Applications, NCSR “Demokritos”, Athens, Greece; ^3^ Shemyakin-Ovchinnikov Institute of Bioorganic Chemistry, Russian Academy of Sciences, Moscow, Russia; ^4^ Institute of Molecular Medicine, Sechenov First Moscow State Medical University, Moscow, Russia; ^5^ PhysBio of MEPhI, Moscow, Russia; ^6^ Department of Pharmacy, University of Patras, Patras, Greece

**Keywords:** nicotinic acetylcholine receptors, α-conotoxins, RgIA, structure, molecular modeling, molecular dynamics

In the published article, there was an error in the **Funding** statement. We failed inadvertently to acknowledge one of our funding bodies in the “**Funding**” section. The **Funding** statement read as “This work was supported by a grant from Stavros Niarchos Foundation (ST and MZ), by RSF (Grant No. 16-14-00215) (VT and II), by RFBR (Grant No. 18-04-01366) (IK) and by BioStruct-X (Contract No. 283570/3521).”

The correct statement is: “This work was supported by a grant from Stavros Niarchos Foundation (ST and MZ), by RSF (Grant No. 16-14-00215) (VT and II), by RFBR (Grant No. 18-04-01366) (IK), by the Hellenic Foundation for Research and Innovation (HFRI) and the General Secretariat for Research and Technology (PG), (StruNic, Grant No. 677) and by BioStruct-X (Contract No. 283570/3521).”

The authors apologize for this error and state that this does not change the scientific conclusions of the article in any way. The original article has been updated.

