# Supplementary material for: Crystal Structure of the Monomeric Extracellular Domain of α9 Nicotinic Receptor Subunit in Complex With α-Conotoxin RgIA: Molecular Dynamics Insights Into RgIA Binding to α9α10 Nicotinic Receptors
Source: Front Pharmacol. 2019 May 1;10:474. doi: 10.3389/fphar.2019.00474 (PMC6504684; doi:10.3389/fphar.2019.00474)
Supplement: Supplementary file 2 [file Data_Sheet_2.docx]

**Supplementary Table 1. Data collection and refinement statistics.**

| Wavelength (Å) | 1.0000 |
| --- | --- |
| Resolution range (Å) | 42.43 - 2.26 (2.341 - 2.26) |
| Space group | P 21 21 2 |
| Unit cell axis (Å) | 63.65 82.53 49.47 |
| Unit cell angles (^o^) | 90 90 90 |
| Total reflections | 67044 (5396) |
| Unique reflections | 12626 (1136) |
| Multiplicity | 5.31 (4.75) |
| Completeness (%) | 99.16 (92.13) |
| Mean I/sigma(I) | 9.9 (1.19) |
| Wilson B-factor (Å^2^) | 46.80 |
| R-merge | 0.1072 (1.315) |
| R-meas | 0.1115 (1.384) |
| R-pim | 0.05621 (0.7429) |
| CC1/2 | 0.997 (0.491) |
| Reflections used in refinement | 12626 (1135) |
| Reflections used for R-free | 631 (57) |
| R-work | 0.1977 (0.3412) |
| R-free | 0.2471 (0.3982) |
| Number of non-hydrogen atoms | 1906 |
| macromolecules | 1824 |
| ligands | 32 |
| solvent | 50 |
| Protein residues | 225 |
| RMS(bonds) (Å) | 0.009 |
| RMS(angles) (^o^) | 1.06 |
| Ramachandran favored (%) | 94.44 |
| Ramachandran allowed (%) | 4.61 |
| Ramachandran outliers (%) | 0.95 |
| Rotamer outliers (%) | 1.49 |
| Clashscore | 11.33 |
| MolProbity validation score | 2.10 (86^th^ percentile) |
| Average B-factor (Å^2^) | 56.76 |
| macromolecules | 57.06 |
| ligands | 54.08 |
| solvent | 47.59 |
| Number of TLS groups | 5 |

*Statistics for the highest-resolution shell are shown in parentheses*


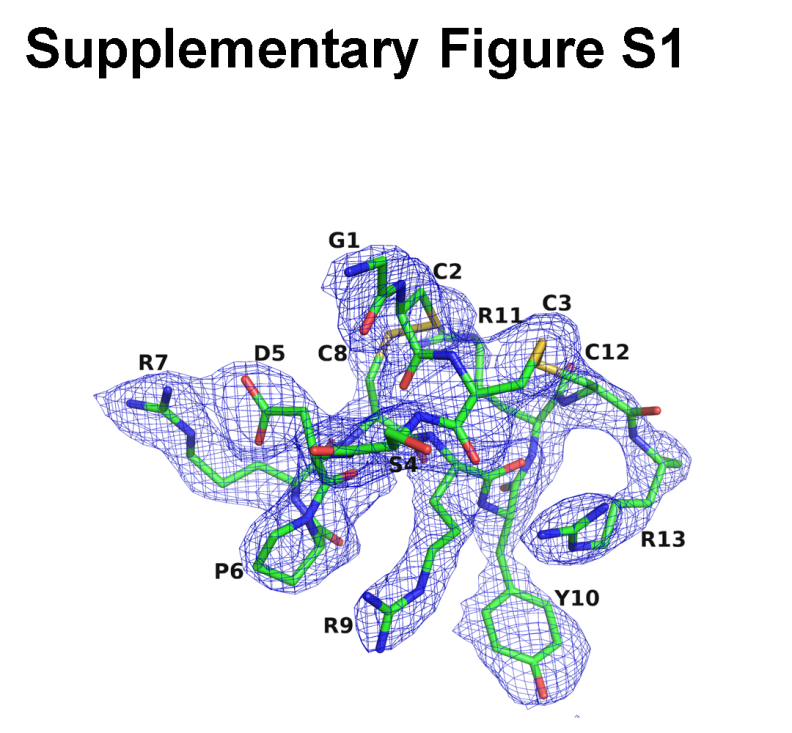


**Supplementary Figure S1 |** The 2F_o_-F_c_ refined electron density map corresponding to the bound RgIA, contoured at 1σ.


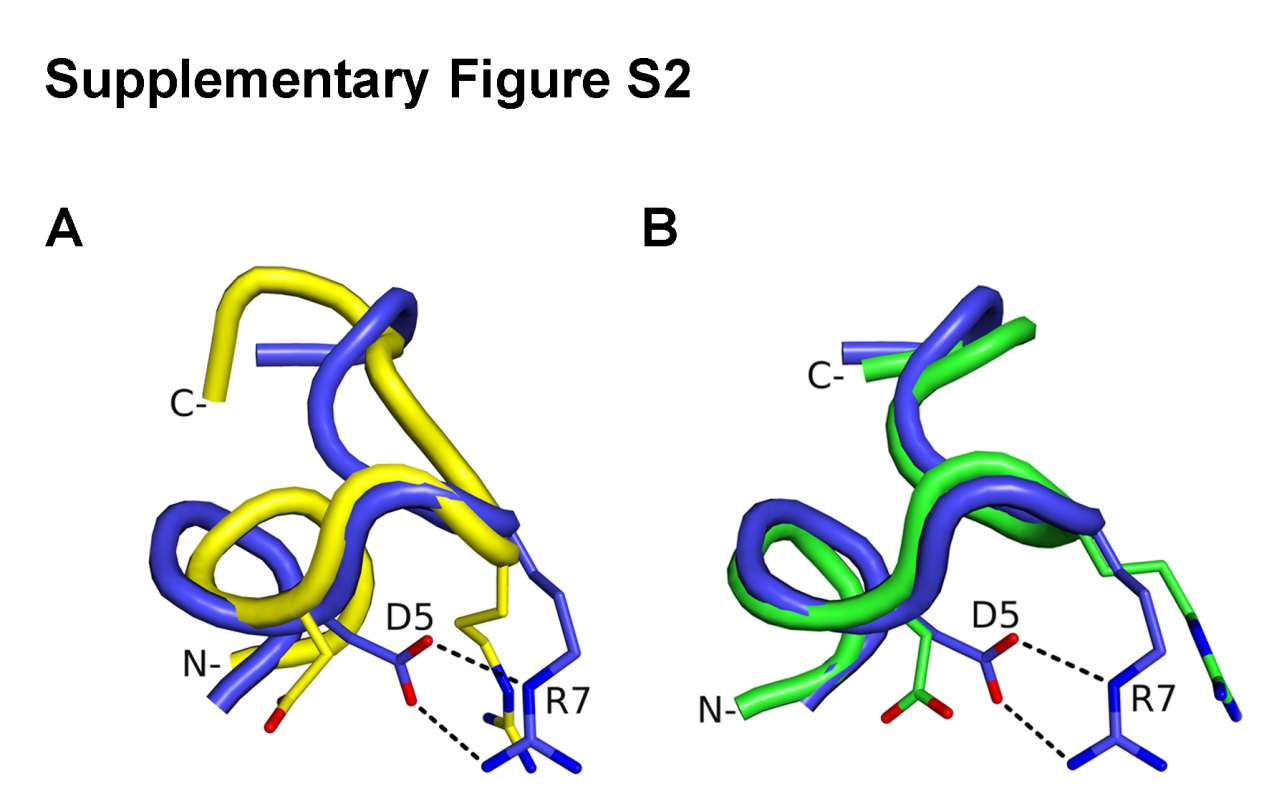


**Supplementary Figure S2 |** Superposition of the crystal structure of α-Ctx ImI (PDB ID: 2C9T) bound to AChBP with its various NMR structures. **(A)** The NMR structure of ImI (PDB ID: 1CNL) (yellow) deviates significantly from its crystal structure (blue), lacking most of the α-helical domain at the middle of the molecule. Moreover, the charged residues Asp5 and Arg7 seem to be non-interacting, contrary to its crystal structure, where they form a strong intramolecular salt bridge. **(B)** Whereas another NMR structure of RgIA (PDB ID: 1IMI) (green) has similar backbone conformation with the crystal structure of ImI (blue), the NMR structure still lacks the intramolecular salt bridge. Notably, this specific charged interaction is present in all crystal complexes of Asp-Pro-Arg triad-containing α-Ctxs.


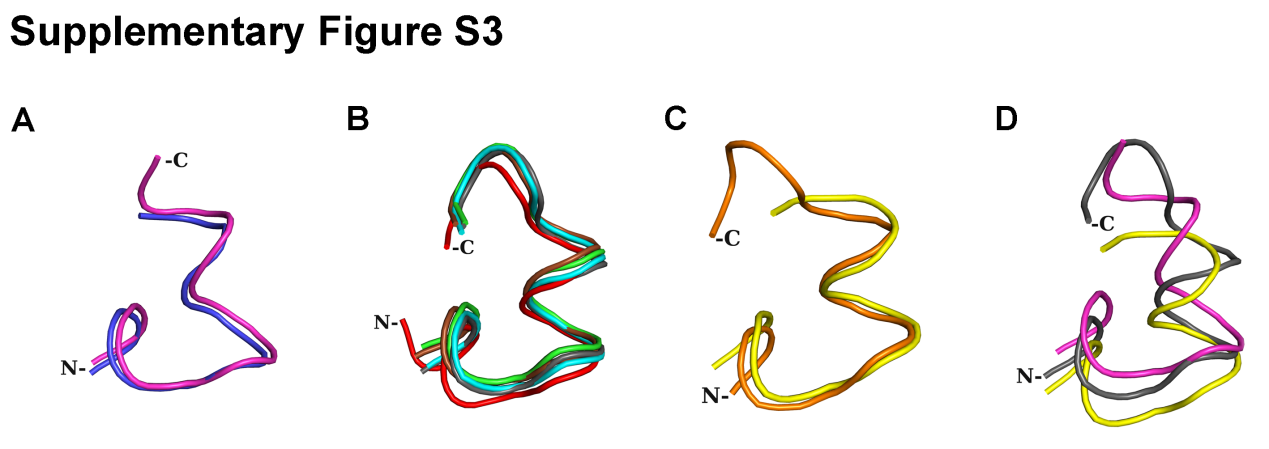


**Supplementary Figure S3 |** Structural clusters of α-Ctxs bound to α9-ECD and homologous proteins. Overlays of RgIA and ImI **(A)**, of GIC, PeIA, PnIA, LsIA and LvIA **(B)**, as well as of BuIA and TxIA **(C)**. **(D)** Superposition of representative α-Ctxs (RgIA, PnIA and BuIA) from each of the three clusters. α-Ctx clustering was produced by superimposing the protomers of AChBPs to the monomeric α9-ECD from their complexes with these α-Ctxs. (RgIA in magenta, ImI in blue, PeIA in cyan, GIC in green, PnIA in grey, LsIA in red, LvIA in brown, BuIA in yellow and TxIA in orange)


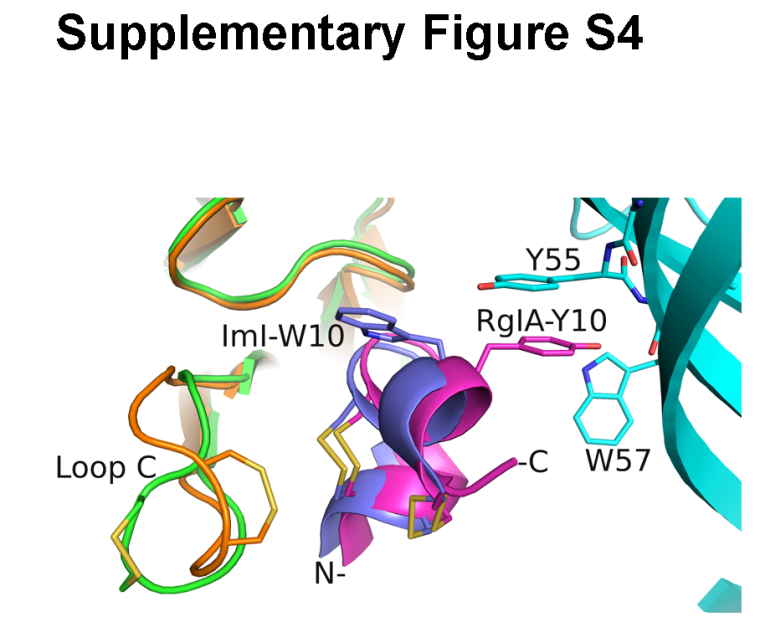


**Supplementary Figure S4 |** Crystal packing contacts. Close side-view of the α9-ECD (green) complex with RgIA (magenta) and of the symmetric adjacent α9-ECD (cyan). Noticeably, the symmetric α9-ECD is in an inverted orientation; thus the two neighboring α9-ECDs do not form a canonical ligand-binding site. As shown by the superposition of the α9-ECD protomer to the protomer of AChBP (orange) with ImI bound (blue), the overall conformation of RgIA and ImI conotoxins is very similar. A notable difference is between the orientation of RgIA-Y10 and ImI-W10, since the former makes crystal packing interactions with the adjacent α9-Y55 and α9-W57.

**Supplementary models of pentameric α9α10 nAChR ECDs with bound RgIA |** Representative energy-minimized structures of equilibrated systems employed in the MD calculations are provided. File α9α10-RgIA_3α92α10_eqmin.pdb contains the pentameric assembly of three α9 and two α10 nAChR ECDs with RgIA bound at the interface of chains D (α9) and C (α10). File α9α9-RgIA_3α92α10_eqmin.pdb contains the pentameric assembly of three α9 and two α10 nAChR ECDs with RgIA bound at the interface of chains B (α9) and A (α9). File α10α9-RgIA_2α93α10_eqmin.pdb contains the pentameric assembly of two α9 and three α10 nAChR ECDs with RgIA bound at the interface of chains D (α10) and C (α9).
